# Supplementary material for: Three delays model applied to pediatric injury care seeking in Northern Tanzania: A mixed methods study
Source: PLOS Glob Public Health. 2022 Aug 25;2(8):e0000657. doi: 10.1371/journal.pgph.0000657 (PMC10021368; doi:10.1371/journal.pgph.0000657)
Supplement: Attachment — Submitted filename: PLOSGlobalPublicHealth_ResponsetoReviewers.docx [file pgph.0000657.s002.docx]

**Response to Reviewers**

Journal Requirements:

**1. Please include a complete copy of PLOS’ questionnaire on inclusivity in global research in your revised manuscript. Our policy for research in this area aims to improve transparency in the reporting of research performed outside of researchers’ own country or community. The policy applies to researchers who have travelled to a different country to conduct research, research with Indigenous populations or their lands, and research on cultural artefacts. The questionnaire can also be requested at the journal’s discretion for any other submissions, even if these conditions are not met.  Please find more information on the policy and a link to download a blank copy of the questionnaire here:** [**https://journals.plos.org/plosone/s/best-practices-in-research-reporting**](https://journals.plos.org/plosone/s/best-practices-in-research-reporting)**. Please upload a completed version of your questionnaire as Supporting Information when you resubmit your manuscript.**

Thank you for this important request. This questionnaire has been completed and uploaded as Supporting Information, and a subsection ‘Inclusivity in Global Research’ has been added to the Methods section of the manuscript to lines 106-110.

**2. Your co-author, Francis Sakita** [**-francis.sakita@gmail.com**](mailto:-francis.sakita@gmail.com)**, has not confirmed authorship of the manuscript. We have resent them the authorship confirmation email; however please check that the above email address for them is correct and follow up personally to ensure they confirm.**

Thank you for letting us know. I have communicated personally with Dr. Sakita and that is the correct email address for him. I have communicated with him and he assures me that he has now confirmed authorship of the manuscript. Please let me know if this is not the case.

**3. We do not publish any copyright or trademark symbols that usually accompany proprietary names, eg (R), (C), or TM  (e.g. next to drug or reagent names). Therefore please remove all instances of trademark/copyright symbols throughout the text, including (C) on page 8.**

Thank you for this correction. All instances of trademark/copyright symbols have been removed throughout the text.

**4. Please update the completed 'Competing Interests' statement, including any COIs declared by your co-authors. If you have no competing interests to declare, please state "The authors have declared that no competing interests exist".**

Thank you for this instruction. This statement has been added to a ‘Competing Interests’ section of the manuscript in lines 632-634.

**5. In the online submission form, you indicated that "Data are available at reasonable request by contacting the corresponding author at** [**elizabeth.keating@hsc.utah.edu**](mailto:elizabeth.keating@hsc.utah.edu)**. We are not able to deposit our data in a public repository given the ethical concerns of sharing qualitative data transcripts.". All PLOS journals now require all data underlying the findings described in their manuscript to be freely available to other researchers, either 1. In a public repository, 2. Within the manuscript itself, or 3. Uploaded as supplementary information.
This policy applies to all data except where public deposition would breach compliance with the protocol approved by your research ethics board. If your data cannot be made publicly available for ethical or legal reasons (e.g., public availability would compromise patient privacy), please explain your reasons by return email and your exemption request will be escalated to the editor for approval. Your exemption request will be handled independently and will not hold up the peer review process, but will need to be resolved should your manuscript be accepted for publication. One of the Editorial team will then be in touch if there are any issues.**

Thank you for making us aware of this. The quantitative registry data has been made freely available in a public repository as instructed and can be found at this url: <https://osf.io/3h95p/> . For the qualitative data, which is interview transcripts, we are unable to post in a public repository due to ethical concerns. Posting the interview transcripts would compromise the confidentiality of our IDI participants as there may be identifying information such as injury mechanisms and types that are unique to the specific patient.

**Review Comments to the Author**
 **Reviewer #1: The study reports analysis of extracted data from 1 year of trauma registry cases at KCMC, Moshi. The authors explored differences in extracted characteristics between those who died or survived to discharge using univariable analysis. It also reports qualitative analysis of 30 conveniently sampled interviews of guardians of injured children mapped to the Three Delays.

I congratulate the authors for addressing an important topic and advancing the application of the Three Delays Model for evaluating injury care health systems.**

Thank you for this compliment.

**I have some comments which I hope are useful to improve the paper.**

Thank you so much for these useful comments that have improved our paper.

**1. The authors report this as a convergent design mixed methods paper. A key tenet of mixed methods research, to my understanding, is that integration of the data / findings occurs to provide understanding / interpretation greater than the sum of its parts. I am not clear how the trauma registry data findings have been integrated with the interview data. Similarly the authors report triangulation across data sources, which to my mind would mean both registry and interview data, but this could be much clearer how these data have triangulated.**

Thank you for this discussion and for pointing out that our integration of the quantitative registry data and qualitative interview data is not clear. In this manuscript, we are identifying time delays using the quantitative pediatric trauma registry. We are then attempting to explain and explore these delays using our qualitative IDIs with caregivers of the patients in our registry. We have attempted to make this more clear in the abstract (lines 29, 31, and 32) and study design section (lines 93-95). Given this clarification, we now feel that our analytic strategy is more appropriately an explanatory mixed methods design, and have replaced convergent with explanatory in the manuscript in line 93. We also removed triangulation of data sources from the abstract and methods line 96 as this was not sufficiently describing our methods. To further demonstrate integration of the quantitative and qualitative data, we have added to the discussion section as well in lines 504 and 510-513.

**2. The variable extraction from the registry could be better reported to explicitly state what variables were extracted. Is the registry on REDCAP or certain variables selected from the registry inputted into REDCAP?**

Thank you for this suggestion. We have added to the Methods ‘Quantitative Procedures’ section in lines 156-160 details including that the registry data were all recorded on REDCap and explicitly stating what variables were extracted from the registry database.

**3. I struggled to follow the approach to analysis of the qualitative data. I find this whole section confusing as to how the anaylsis was conducted and by whom at which stage and how the iterations occurred. A flow diagram could perhaps clarify. Further, who wrote the memo and how? Was this written by the team as a team based approach? Condensing an hour interview to 2 sides represents a significant initial analysis to filter / code the data and who did this is important to understand.**

Thank you for requesting clarification on the qualitative analysis section. This section has been clarified with additional details on who performed each step of the qualitative analysis and memo writing process. Additionally, a reference on memo writing (28) in qualitative research has been added to strengthen and support our approach. This text has been added to lines 213-223: “De-identified data were analyzed through a team-based, thematic approach informed by applied thematic analysis with qualitative memo writing.[27, 28] After multiple readings of the IDI transcripts, a memo summarizing each transcript was written by investigator BY after training by EMK and MHW. Memos followed an established template of a priori domains, informed by the interview guide, to extract and synthesize the text’s core meaning related to the research questions and to extract representative quotes. The memo-writing process identified emerging themes and informed the development of preliminary codebooks. Each memo was on average two single-spaced pages long and reviewed by EMK to ensure that the interview content was being interpreted appropriately. After the memos were reviewed, investigators BY, EMK, MHW, and SR met to reach team consensus on the emerging themes and finalize the codebook. The memos were then coded in Dedoose software, and the codebook was continually adapted to reflect new or emerging themes as coding progressed.”

**4. I would like to understand how the convenience sample was performed. The authors report 30 hours of interviews over 12 months from 2 interviewers (possibly 15 hours each). Can you elaborate why it took so long and in what way the sample was convenient?**

Thank you for this request. More information on how the convenience sample was performed has been added to lines 145-147: “Attention was given to interviewing caregivers who would be good informants. In order to adequately represent our patient population, we attempted to interview caregivers of patients with a variety of ages, injury mechanisms, and injury types.” Our research assistants performed the IDIs slowly over the course of the 12 month study period, approximately 1 per week or every other week. Completion of all 30 of the IDIs took so long because our research assistants were also performing quantitative data collection and translation/transcription of the IDIs during the 12 month study period.

**5. Univariable analysis was performed. I was a little confused as to how the analysis generated 4 p values for the mechanism of injury categorical variable but only 1 for the other categorical variables such as transport type and initial facility. I wondered whether a multivariable logistic regression analysis might have better summarised the associations between death and the variables explored, whilst controlling for confounders.**

Thank you for these questions regarding our analysis. The mechanism of injury categorical variable had 4 P values because a child sometimes had more than one mechanism of injury, such as if they were struck and then fell or if they were hit by a motor vehicle and had a burn from the exhaust pipe (this happened a number of times). To eliminate confusion we have added an * at the bottom of Table 1 to explain that children could have more than one mechanism of injury. We performed only a univariate and not a multivariable model given the low number of deaths in our sample.

**6. If you do chose to use univariable categorical analysis where sizes are n<5 I think fisher’s exact should be used instead of chi squared. And a correction for multiple hypothesis testing e.g. Bonferroni, would be appropriate)**

Thank you for these suggestions. We have updated the cells in Table 1 using Fisher’s exact test, and added this to the quantitative analysis section of the Methods. We do not feel that a Bonferroni correction is appropriate in this case because our analysis is preliminary and is presented as such; therefore, no adjustment for multiple testing was made. A reference supporting this decision can be found here: <https://www.annalsthoracicsurgery.org/article/S0003-4975(15)01873-1/fulltext>.

**7. The authors have conceived of interfacility delays within the third delay. Other researchers have categorised this as within the third delay (DOI:**[**https://doi.org/10.1016/S2214-109X**](https://doi.org/10.1016/S2214-109X)**(20)30067-X) You may wish to reflect on this categorisation in the discussion.**

Thank you for this comment. I am guessing you meant that we conceived of interfacility delays within the second delay. Yes, other researchers have categorized this differently. However, other researchers have also categorized this as we have. We chose to categorize them as within the second delay because we chose to count receipt of care (Delay 3) as care received *specifically at KCMC*, where the study was conducted. We have now added clear definitions of each delay to the Methods section in lines 227-249.

**8. I found the narratives selected to be helpful and powerful illustrations of the issues summarised.**

Thank you for this comment; we are glad the selected narratives are powerful.

**9. When studying / discussing delay 3 I think the authors could have a broader understanding of delay 3 also encompassing the quality / appropriateness of care. Only considering purely a time delay to ED assessment will not capture anything about the quality of this ED assessment and the process a of the care delivered and the outcome for the patient. This should at least be acknowledged in the limitations.**

Thank you for this important suggestion. You are correct that our study addresses the time delay in receiving appropriate care as Delay 3, but does not measure quality of care received. We agree that this is a limitation of our study, and we have added to the limitations section in lines 603-611: “In addition, we defined Delay 3 as delays in reaching appropriate care at KCMC, but we did not capture the quality of care received. Simply reaching appropriate care at KCMC does not equal high quality of care, and thus this is a limitation.”

**Minor points:**
**10. Ref 13 makes the point that the time critical nature of trauma is variable and the importance of timeliness depends of the nature of injury. This nuance should perhaps be reflected if using this reference.**

Thank you for this important point and addition. To add this nuance on the time critical nature of trauma being most important in severely injured children we have added “especially in those with severe injuries” to line 66 in the Introduction.

**11. Registry inclusion and exclusion criteria descriptions are repetitive (lines 114 – 117).**

Thank you for pointing this out. The repetitive section of the exclusion criteria has been deleted.

**12. Money was provided for transportation costs. Did the caregivers have to travel to conduct the interviews? this is not clear.**

Thank you for this request for clarification. No, the caregivers did not have to travel to conduct the interviews. We worded the reimbursements that we provided the caregivers as for “time and transportation costs” as many caregivers did use the funds for transportation costs home after discharge from the hospital. However, they were mainly to compensate their time, so “transportation costs” has been removed.

**13. The abbreviation PI should be in full at first usage.**

Thank you for this comment. PI was changed to Principal Investigator at first usage in line 157.

**14. Figure 2 labelling should reflect that it is both survivors and deaths being demonstrated.**

Thank you for this suggestion. We have changed the Figure 2 title and legend to better reflect that there are both survivors and those that died being demonstrated.

**15. The first line of the discussion refers to care seeking delays, which is delay 1. But I think you’re referring to applying the 3 delays model to analysing paediatric injury care in an LMIC?**

Thank you for catching this. Yes, you are correct that we are not referring to care seeking only (Delay 1). “Seeking” has been removed from the first sentence of the Discussion so that the sentence applies to all 3 delays rather than just delay 1.

**16. The authors use the term intermediary to refer to facilities other than KCMC. Presumably these other hospitals are not always intermediary but rather primary and secondary care that can manage many things independently but need to refer to tertiary care for some conditions. I'm not sure intermediary is the correct/most helpful term.**

Thank you for this important point. You are correct that these other hospitals are not always intermediary and are often primary and secondary. For our purposes in this manuscript, however, these facilies are intermediary since all of our patients were transferred to tertiary care at KCMC. When referring to these hospitals in the context of this manuscript, we have chosen to keep intermediary. When referring to outside hospitals in the generalized broader application and not in the context of this study, we have changed from “intermediary” to “outside”. We have made these changes in the discussion and limitations sections in lines 536, 541, 593, 597, and 598.

**17. The first discussion paragraph refers to interventions. But I think your findings are identifying barriers rather than the solutions.**

Thank you for this comment. We have clarified that we have identified barriers that could be addressed with interventions rather than identifying the interventions themselves by adding text to the last sentence of the first discussion paragraph in lines 512-513 so that it now reads “…identified barriers that contribute to delays along the care continuum. These barriers could be addressed with interventions including…”.

**18. Line 436 – I don’t think education on primary prevention of injury will influence care seeking after injury has occurred.**

Thank you for catching this. “Primary prevention of injury” has been removed from this sentence now in line 527.

**19. When discussing mortality rates (line 464) you may wish to reflect on the long pre KCMC timelines that function to pre select surviving patients who have actually made it to tertiary care.**

Thank you for this comment. We do reflect on this in the limitations section, and have added the following to this reflection in lines 592-594: “We are likely missing some children who presented to intermediary facilities for care and never made it to KCMC due to the long pre-KCMC timelines that function to pre-select surviving patients.”

**20. In 470 increased enrolment in health insurance is discussed. This reads like such a system exists in Tanzania but is not commonly used. Is such a system present or are you proposing one should be developed? Or does this national insurance mean a taxpayer funded health system free for all to access?**

Thank you for these questions. Yes, national health insurance does exist in Tanzania but is not commonly used due to cost of enrollment. The percentage of our population enrolled in insurance was added to the results, and details on this were added to the Discussion to lines 558-559 and 571-572.

**21. In limitations you may wish to reflect on the lack of injury severity variable from the registry reported to benchmark care quality performance with elsewhere / other standards.**

Thank you for this suggestion. We have added the following sentence on injury severity to the limitations in lines 612-614: “Further, we did not include a variable on injury severity in the registry which could help to benchmark care quality performance with other settings.”

**22. I don’t think reference 37 is properly represented. This reference study reported which of the three delays studies in the published literature have focused on. The authors own study is a rarity focussing on all three delays. Most of the published trauma care literature in LMCs is facility centric. Reference 37 is not really in contrast to this study’s findings.**

Thank you for this comment. We agree with you and apologize for misrepresenting reference 37. We have edited this paragraph to better represent the study in lines 577-583 by removing “In contrast to our findings,” and adding the following sentence, “As the majority of the published trauma literature in LMICs is facility-centric, our study is a rarity by reporting on all three delays.”

**23. Re line 494 - perhaps explain social desirability and how that would have specifically potentially affected your findings.**

Thank you for this suggestion. We have added the suggested information by adding this sentence to lines 601-603: “This social desirability bias could have made caregivers less comfortable in discussing delays in receiving care at KCMC, which could be the reason we did not identify as many themes in this delay.”

**24. In figure 1 the regional and district hospitals are separately shown in the flow diagram but jointly analysed in the timeline to care arrows below. Perhaps be consistent**

Thank you for this suggestion. Since the two levels of hospitals were analyzed together we have changed Figure 1 to be consistent with this.

**Reviewer #2: Thank you for the opportunity to review this manuscript on the three delays model as applied to pediatric injury in Tanzania.**

Thank you for reviewing our manuscript.

**One of the biggest challenges with discussing delays is being very precise on what is defined as a delay. To have a "delay" you would need to have set standard expectations in which a patient/caretaker should seek, reach, or receive care and this would likely vary based on the injury and severity. In this study, delay is not specifically measured or defined. Rather, this study reviews important factors which can influence delays and captures data from caretakers on perceived delays.**

Thank you for this discussion on the challenges of discussing delays. We agree that our study does not specifically measure or define a delay, but rather reviewers factors that influence delays and captures the caregiver perspective on perceived delays.

**25. To better understand the context, it would be helpful to have more detail on the study setting. For example, is there an ambulance or EMS service? Are there pediatric surgeons at KCMC? What other services or healthcare facilities are available in the region? is this the only regional hospital with pediatric services? Is there a burn center at KCMC? What is the catchment area for KCMC? Each of these factors may play into the referral patterns and therefore potential delays.**

Thank you for these important questions. More context on the study setting has been added to the Study Setting section of the Methods in lines 115-117: “The study took place at Kilimanjaro Christian Medical Centre (KCMC), a zonal referral hospital for the northern regions of Tanzania (Arusha, Dodoma, Kilimanjaro, Singida, and Tanga). KCMC is one of four zonal referral hospitals in Tanzania, and has a catchment population of 12,000,000 persons.” In addition, in lines 120-123 the following details were added: “KCMC has general surgeons and orthopedic surgeons who treat trauma patients, including children. In addition, KCMC has pediatricians that assist in the care of pediatric trauma patients. There is a specialized burn center at KCMC. There is no formal emergency medical system in Tanzania.”

**26. In the quantitative analysis, did you capture data on severity of injury?**

Thank you for asking about severity of injury. No, we did not capture severity of injury in the quantitative analysis for this manuscript. This has been added as a limitation to the limitations section as described above in #21.

**27. Was there any association between the demographic characteristics and delays? Is injury mechanism or illness severity associated with delays?**

Thank you for asking these questions. We did not specifically look at any association between demographics or injury mechanism and delays. As mentioned above, we did not capture illness severity in this manuscript, though this is a very good point since severity is important when talking about delays. A future project is currently in progress that will aim to examine this.

**28. Some of the columns in table 1 total more than 100%. For example, the mechanism of injury totals 134.8%(37.5+11+34.2+52.1).**

Thank you for this question. The mechanism of injury categorical variable totals greater than 100% because a child sometimes had more than one mechanism of injury, such as if they were struck and then fell or if they were hit by a motor vehicle and had a burn from the exhaust pipe (this happened a number of times). To eliminate confusion we have added an * at the bottom of Table 1 to explain that children could have more than one mechanism of injury.

**29. The first paragraph on the quantitative results (page 13, lines 213-218) describing the delays should be in the methods section rather than results.**

Thank you for this suggestion. I assume you mean the first paragraph on the qualitative results, as these are listed on page 13, lines 213-218. We have moved these lines to the methods section (lines 227-249) as you have suggested.

**30. As the quantitative analysis was conducted on a convenience sample, how can you be sure that this is representative of the patient population? Were patients who died included in the quantitative analysis? What were the characteristics of the patients selected for IDI?
Some of the themes were identified in only a couple of examples (money to pay bills, wait time). Is this really a theme if only 2-3 patients had this experience?**

Thank you for these questions. We feel that the convenience sample was representative of our population, as we tried to select caregivers of patients with varying ages, injury mechanisms, and injury types. Patients who died were included in the quantitative analysis (mortality rate 8.2% was reported in our manuscript). We assume you are actually asking about the qualitative analysis, however, and one caregiver of a patient who subsequently died was included in the qualitative IDIs. We did not feel that it was ethical to approach a caregiver of a patient who had died immediately on arrival to KCMC and request an interview, so all of our interviews were with caregivers of patients who survived the initial stabiliziation and admission to the hospital. We have added to the limitations section (lines 614-616) that caregivers of patients that had immediate mortality at KCMC were not included in the interviews due to these ethical and logistical concerns. Regarding the themes identified in only a couple of examples, we felt that given the variability of this patient population with many different ages, injury mechanisms, and injury types, something that emerged across two or more interviews warranted discussion as a theme. Although this may not have been a barrier to all, it was a barrier to some, and we felt that these warranted discussion.

**31. Was there any data captured on actual times for each level of delay? It seems that this data is really on patient and caretaker perceived delays, rather than actual medical delays. For example, there are different time expectations in regards to management of an open fracture compared with a closed fracture. Without this level of detail, it may be difficult to accurately capture data on delays.**

Thank you for this comment. Yes, we did capture data on the actual times for each level of delay. Our injury registry captures time of injury, time of first care, and time of arrival to KCMC, and this information is included in this manuscript. We have included that these variables are included in the Methods ‘Quantitative Procedures’ section to make this more clear in lines 158-160: “**For the purposes of this analysis,** variables were extracted from the REDCap database regarding: demographics, mechanism of injury, time of injury, mechanism of transport, first health facility treated at, time of first care, time of arrival to KCMC, and patient outcomes including in-hospital mortality.”

**32. In the discussion, you state that "delaying presentation to definitive care in children that need transfer is associated with a higher mortality risk (page 24, line 447-448)." I'm not sure that you can make that conclusion in this study. You do not know the mortality associated with patients who were not transferred (and managed at the other sites). Also, patients who were transferred may have had higher injury severity and therefore other factors which contributed to mortality.**

Thank you for these comments. We agree with your concern that we cannot make that conclusion in this study. Thus, we have changed/softened the language of this sentence to read in line 538: “…in children that need transfer may confer greater risk for mortality”. In addition, we added to the limitations that we do not know the mortality associated with patients who were not transferred, and also that we did not include a measure of injury severity.

**33. Similarly, later in the same paragraph, you state that "intermediate facility healthcare workers may not know when to refer severely injured patients (page 24, line 451)." Again, you don't show data on the patients managed at the intermediate facility and you don't show data on illness severity.**

Thank you for this comment. While it is true that we do not show data on the patients managed only at the intermediate facility and don’t have data on illness severity, we do have qualitative data that suggests the healthcare workers might not know when to refer patient’s to KCMC (specifically the quote on page 22 lines 452-460). However, to address your valid concern, the following sentence was added to the limitations in lines 596-598: “In addition, we did not collect quantitative or qualitative data at the intermediary facility level so we are missing both outcomes data and perspectives of patients who were not transferred.”

**34. You comment on the need for increased enrollment in health insurance. What percentage of this study population had health insurance?**

Thank you for this question. 22.62% of our study population had health insurance, and this has been added to the Results section to lines 258-259.
